# MICS/ISM Meander-Line Microstrip Antenna Encapsulated in Oblong-Shaped Pod for Gastrointestinal Tract Diagnosis

**DOI:** 10.3390/s21113897

**Published:** 2021-06-04

**Authors:** Supakit Kawdungta, Akkarat Boonpoonga, Chuwong Phongcharoenpanich

**Affiliations:** 1 Faculty of Engineering, Rajamangala University of Technology Lanna, Chiang Mai 50300, Thailand; supakitting@rmutl.ac.th; 2 Faculty of Engineering, King Mongkut’s University of Technology North Bangkok, Bangkok 10800, Thailand; akkarat.b@eng.kmutnb.ac.th; 3 School of Engineering, King Mongkut’s Institute of Technology Ladkrabang, Bangkok 10520, Thailand

**Keywords:** dual-band, flipped-L, gastrointestinal tract, meander-line, specific absorption rate

## Abstract

In light of the growth in demand for multiband antennas for medical applications, this research proposes a MICS/ISM meander-line microstrip antenna encapsulated in an oblong-shaped pod for use in diagnoses of the gastrointestinal tract. The proposed antenna is operable in the Medical Implant Communication System (MICS) and the Industrial, Scientific and Medical (ISM) bands. The antenna structure consists of a meander-line radiating patch, a flipped-L defected ground plane, and a loading resistor for antenna miniaturization. The MICS/ISM microstrip antenna encapsulated in an oblong-shaped pod was simulated in various lossy-material environments. In addition, the specific absorption rate (SAR) was calculated and compared against the IEEE C95.1 standard. For verification, an antenna prototype was fabricated and experiments carried out in equivalent liquid mixtures, the dielectric constants of which resembled human tissue. The measured impedance bandwidths (|*S*_11_| ≤ −10 dB) for the MICS and ISM bands were 398–407 MHz and 2.41–2.48 GHz. The measured antenna gains were −38 dBi and −13 dBi, with a quasi-omnidirectional radiation pattern. The measured SAR was substantially below the maximum safety limits. As a result, the described MICS/ISM microstrip antenna encapsulated in an oblong-shaped pod can be used for real-time gastrointestinal tract diagnosis. The novelty of this work lies in the use of a meander-line microstrip, flipped-L defected ground plane, and loading resistor to miniaturize the antenna and realize the MICS and ISM bands.

## 1. Introduction

Recent decades have witnessed rapid growth in the adoption of wireless communications technologies in the medical domain, especially those operable in the microwave frequency band [1,2,3,4,5,6,7,8]. In the medical field, wireless technology currently has four applications: therapeutic, sensing, imaging, and telemedicine applications. The therapeutic operating frequency is predominantly for remedial and diagnostic purposes, such as cardiac ablation and cancer therapy [1,2,3]. The sensing and imaging frequency bands are used in in vitro and in vivo diagnoses and magnetic resonance imaging (MRI) [1,2,3], and the telemedicine frequency is for health-care provision and medical consultation [4,5,6,7,8].

There are four universal microwave frequency bands for wireless communications in the health and medical domains: the Medical Implant Communication System (MICS, 401–406 MHz); the Wireless Medical Telemetry Service (WMTS, 1427–1432 MHz); the Wireless Body Area Network (WBAN, 2360–2400 MHz); and the Industrial, Scientific and Medical (ISM) bands (2.4–2.5 GHz) [8,9,10]. In practice, medically implantable and/or ingestible antennas should be small, operable at the medical frequency bands, and harmless to humans.

In [11], a 3D-spiral small antenna operable in the MICS band with a 225.5 MHz bandwidth was proposed for biomedical telemetry. The antenna structure consists of a spiral short-circuit, folded rectangular patch on a double-layer substrate (Teflon and ceramic) with dimensions of 14 × 14 × 15 mm (*W* × *L* × *H*). In [12], the authors proposed an implantable slot dipole conformal antenna operable in the ISM bands, embedded in biocompatible polydimethylsiloxane (PDMS) for total size reduction. The specific absorption rate (SAR) of the antenna in the human muscle tissue liquid was also calculated.

In [13], a planar inverted-F antenna (PIFA) was proposed to enhance the antenna bandwidth in the MICS band. The proposed PIFA can achieve 120 MHz bandwidth and is operable in the 353–473 MHz frequency band. A compact broadband antenna using a triple-layer substrate has been proposed for the MICS band (402 MHz), and the antenna can achieve an impedance bandwidth of 50 MHz [14]. The aforementioned antennas [11,12,13,14] are single-band antennas for medical applications.

Modern medical applications nevertheless demand two or more frequency bands. As a result, a dual folded dipole antenna fed with a coplanar waveguide, with dimensions of 25 × 34 × 2.5 mm, has been proposed [15]. This antenna can achieve impedance matching in the WMTS (1.4–1.43 GHz) and ISM (2.4–2.48 GHz) bands. In [16], an anti-spiral antenna fed with an L-shaped transmission line, operable in the MICS and ISM bands, was fabricated using a Rogers 3210 substrate (dielectric constant of 10.2), with dimensions of 15 × 15 × 1.92 mm. A tri-band slot PIFA, operable in the MICS, WMTS, and ISM bands, was reported to achieve bandwidths of 16 MHz, 100 MHz, and 200 MHz, respectively [17]. The tri-band antenna is comparably bulky and suffers from transmission overburden. Nonetheless, multiband antennas are better for medical applications than single-band ones.

In addition, the electromagnetic properties of the surrounding environment have an effect on the in-body antenna characteristics. In [18], the analytical results for a Hertz dipole embedded in a lossy multilayer spherical body were presented. The dyadic theory of Green’s function was employed. The proposed method can be used for in-body antennas with spherical boundaries. A low-profile, conformal microstrip antenna has been proposed for in-body capsule applications [19]. This proposed conformal antenna is operable at the center frequency of 434 MHz, with a bandwidth of 16 MHz. The experimental results validated the impedance detuning caused by the high permittivity of the surrounding biological environment. In [20], the stepped-impedance resonator (SIR) technique and dielectric-loaded with ceramic shell and pure water inner filling were employed to miniaturize the antenna. It was simulated in a body model containing nine dispersive tissues. Detuning effects were found in this proposed antenna. An inward-directed antenna for the gastrointestinal, operable at a center frequency of 2.45 GHz, has also been investigated. The antenna characteristics were analyzed at different locations in the torso [21].

This research thus proposes an MICS/ISM meander-line microstrip antenna encapsulated in an oblong-shaped pod for medical applications, specifically for use in diagnoses of the gastrointestinal tract. The proposed microstrip antenna is operable in the MICS and ISM bands and its structure consists of a meander-line radiating patch, a flipped-L defected ground plane, and a loading resistor for antenna miniaturization. The MICS/ISM microstrip antenna encapsulated in the pod was simulated in different lossy-material environments: in a pseudo-muscular cubicle, a multilayer spherical body-parts model, and a quasi-human body. The specific absorption rate of the quasi-human body was determined and compared against the IEEE C95.1 standard. For validation, an antenna prototype was fabricated and experiments carried out in equivalent liquid mixtures, the dielectric constants of which resembled human tissue. The advantages of the proposed meander-line microstrip antenna include its dual-band operation (MICS/ISM bands), relatively small size, and low cost due to the use of one single layer of substrate. The proposed meander-line microstrip antenna could be encapsulated in an oblong-shaped pod for use in diagnoses of the gastrointestinal tract. Table 1 compares the physical dimensions and gain levels of existing multiband antenna schemes for medical-related applications and those of the proposed meander-line microstrip antenna. The dimensions of the proposed meander-line microstrip antenna, 6 × 28 × 1.27 mm (*W* × *L* × *H*), are considerably smaller than those of existing antenna schemes.

## 2. Camera Pod Scheme and Antenna Structure

**Figure 1:**
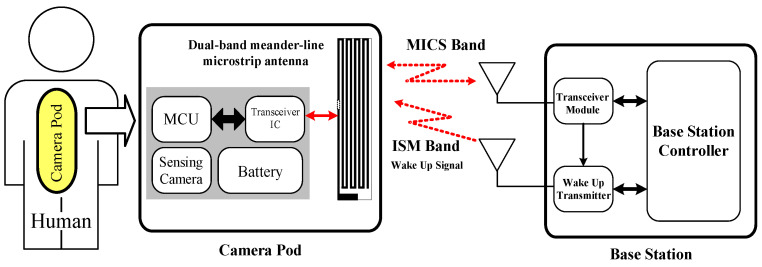
The MICS/ISM meander-line microstrip antenna encapsulated in an oblong-shaped pod, together with the base station.

Figure 1 depicts the MICS/ISM meander-line microstrip antenna encapsulated in a glass pod (22 × 32 mm in ∅ and length) and the base station. The oblong-shaped pod contains the proposed MICS/ISM microstrip antenna, a sensing camera, a microcontroller unit (MCU), and a button cell battery. The pod is designed for intestinal tract diagnosis with minimal damage to the small intestines, which is prone to occur with conventional gastrointestinal tract radiography. As the sensor is used in vitro, the glass pod will not be broken. Besides, the focus of the study is the performance of the MICS/ISM meander-line microstrip antenna.

In the diagnosis, a patient orally ingests a pod which courses through the digestive tract and is excreted with the feces. The sensing camera captures images inside the gastrointestinal tract and the data are transmitted in real-time using the MICS band. Specifically, data are transmitted using the MICS band and the ISM bands are used to activate the pod from sleep mode. Sleep mode was incorporated to ensure efficient battery usage, since it usually takes 6 h after ingestion for food intake to reach the small intestine.

**Figure 2:**
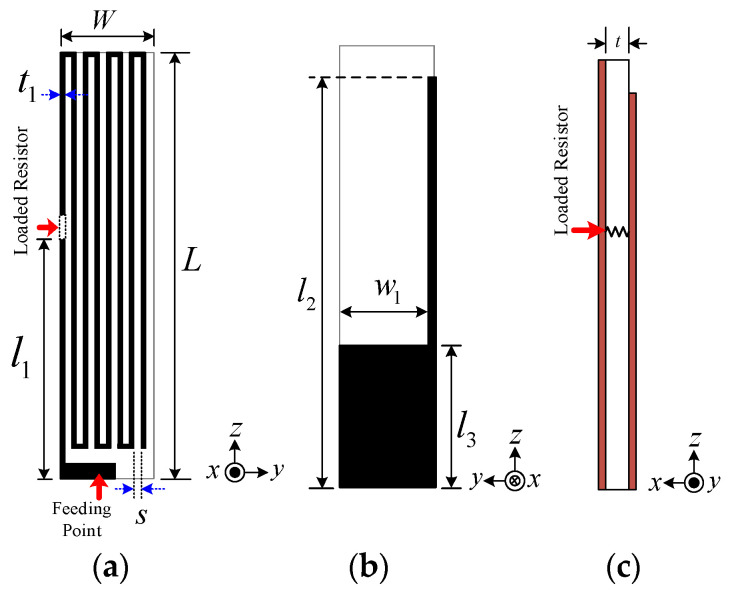
Structure of the MICS/ISM meander-line microstrip antenna: (**a**) front view, (**b**) rear view, (**c**) side view.

Figure 2 depicts the geometry of the proposed MICS/ISM meander-line microstrip antenna. A loading resistor, connecting the meander-line radiating patch with the flipped-L defected ground plane, was used to miniaturize the antenna. In [22,23,24,25], a microstrip antenna with a shorted pin connected to the ground plane was able to reduce the size of the antenna to a quarter-wavelength, and the shorted pin loading (resistor or capacitor) could further decrease the size to less than a quarter-wavelength due to the shift of the resonant frequency of the fundamental mode. The proposed antenna was implemented on a printed circuit board (PCB) with sheets of insulating material (substrate layer) and copper (metal layers) on the top and bottom sides. A PCB of Roger RO3210 substrate material was employed, which has a dielectric constant of 10.2, thickness of 1.27 mm (with copper cladding of 35 μm), surface resistivity of 10^3^ MΩ, and loss tangent (tan δ) of 0.0027 [26]. The notations of the antenna parameters are defined in Table 2.

The antenna can achieve the impedance matching (|*S*_11_| ≤ −10 dB) at 400 MHz and 2.4 GHz with a quasi-omnidirectional radiation pattern. The first resonance-matching (MICS band) is attributable to the meander line and the second resonance (ISM bands) to the flipped-L defected ground plane. Since the pod with the MICS/ISM antenna encapsulated is intended for use inside the human body (i.e., a lossy material), simulations were carried out in different lossy-material environments: a pseudo-muscular cubicle, a multilayer spherical body-parts model, and a quasi-human body.

## 3. Results and Discussion

### 3.1. Simulation Results

The MICS/ISM microstrip antenna encapsulated in an oblong-shaped pod was simulated using CST microwave studio [27] in various lossy-material environments: a pseudo-muscular cubicle, a multilayer spherical body-parts model, and a quasi-human body.

**Figure 3:**
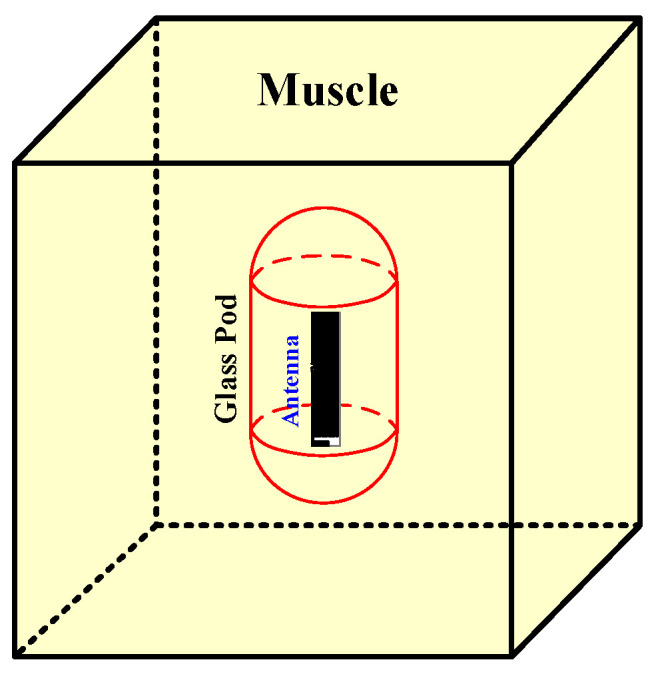
The MICS/ISM meander-line microstrip antenna encapsulated in a glass pod inside the pseudo-muscular cubicle.

Figure 3 depicts the MICS/ISM microstrip antenna encapsulated in a glass pod inside the pseudo-muscular cubicle (50 × 50 × 50 mm). There was air between the antenna and the container. Therefore, the material around the antenna and glass pod was investigated. The muscular cubicle was of lossy material with very high dielectric constants (57.12 and 52.72 for MICS and ISM, respectively). The simulation was carried out in the cubical model to ensure straightforwardness and time efficiency vis-à-vis the quasi-human body [12].

**Figure 4:**
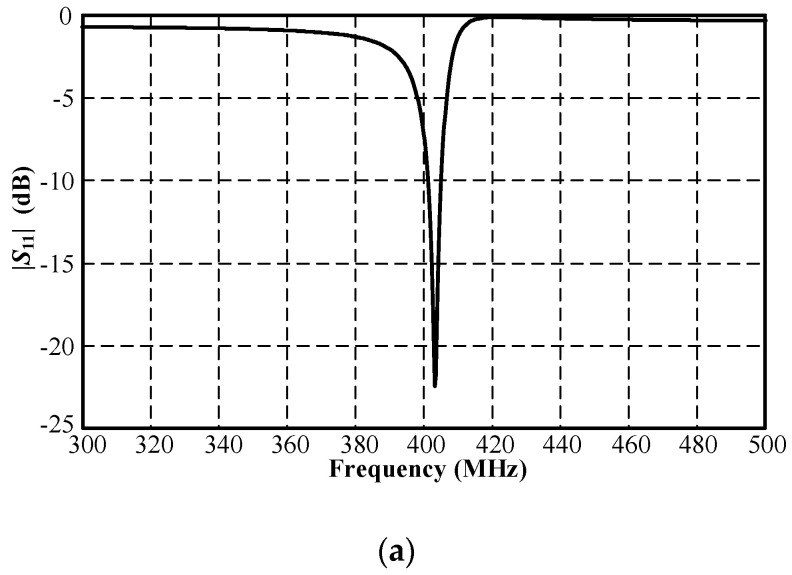

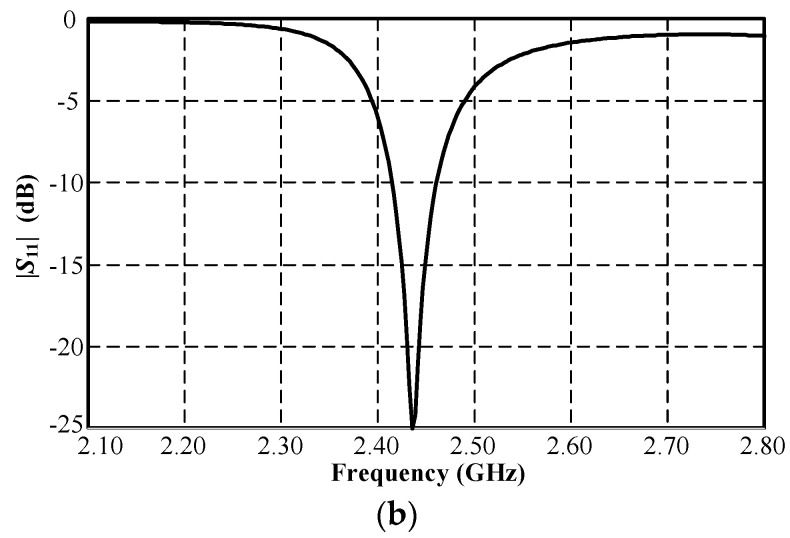
Simulated |*S*_11_| of MICS/ISM meander-line microstrip antenna encapsulated in a glass pod inside the muscular cubicle: (**a**) MICS and (**b**) ISM bands.

**Figure 5:**
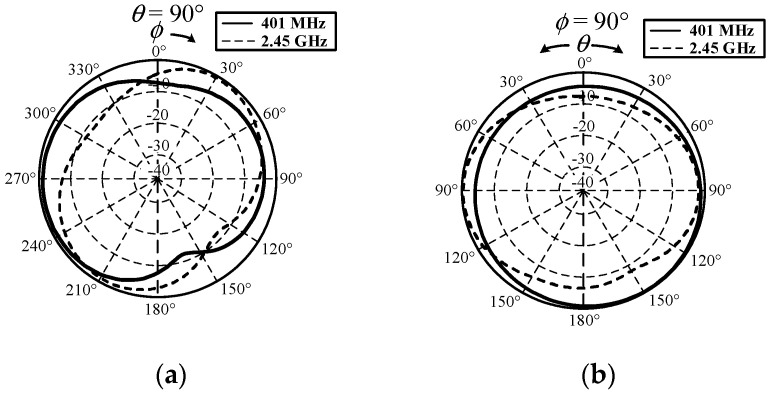
Simulated radiation pattern of MICS/ISM meander-line microstrip antenna encapsulated in a glass pod inside the muscular cubicle: (**a**) *xy* and (**b**) *yz* planes.

**Figure 6:**
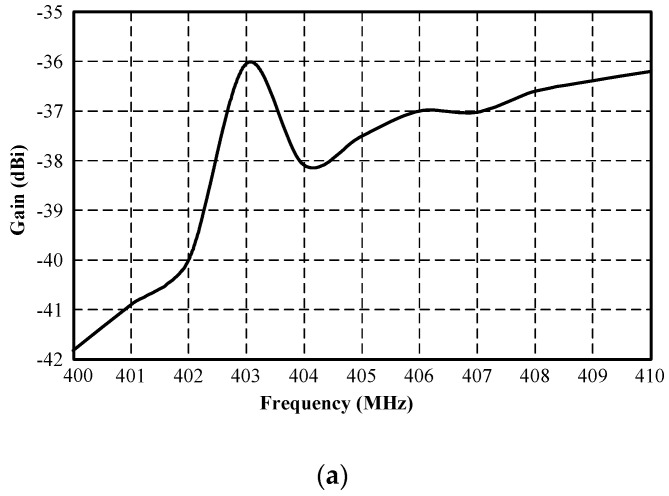

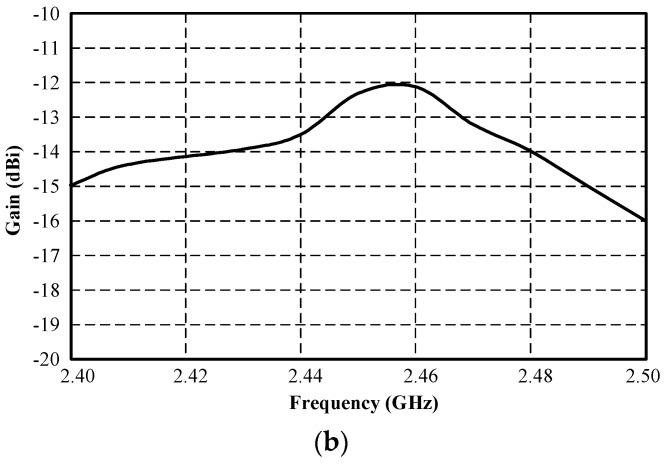
Simulated gain of MICS/ISM meander-line microstrip antenna encapsulated in a glass pod inside the muscular cubicle: (**a**) MICS and (**b**) ISM bands.

The simulation inside the muscular cubicle was performed to determine the initial physical size of the antenna, as shown in Table 2. The simulated impedance matching (|*S*_11_| ≤ −10 dB) was achieved at 400–406 MHz and at 2.41–2.47 GHz, as shown in Figure 4. Figure 5a,b respectively show the simulated *xy*- and *yz*-plane radiation patterns of the antenna in the muscular cubicle. The antenna gains at 403 MHz and 2.45 GHz (center frequency) were −36.04 dBi and −12.31 dBi, respectively. Moreover, the antenna gains along the operating frequency of the MICS/ISM meander-line microstrip antenna encapsulated in a glass pod inside the muscular cubicle are shown in Figure 6a,b.

**Figure 7:**
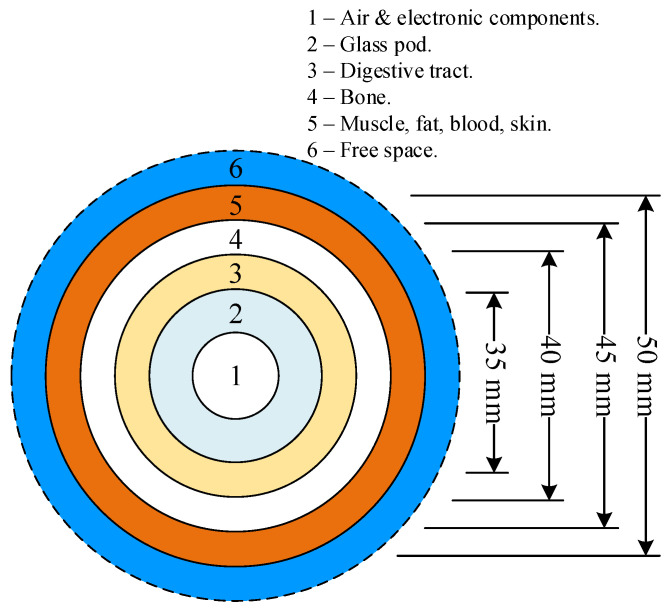
The lossy multilayer spherical body-parts model with varying dielectric constants.

Figure 7 depicts the lossy multilayer spherical body-parts model with the MICS/ISM microstrip antenna encapsulated in a glass pod at the innermost circle (area 1) [10,18]. In the simulation, the six layers of the lossy multilayer spherical body-parts model represented human internal organs and body parts with varying dielectric constants (Table 3) [28]: layer 1 was air, layer 2 was the glass pod, layer 3 was the stomach, layer 4 was the bone, layer 5 was the muscle, and layer 6 was free space. The dielectric constant plays a crucial role in the data-transmission performance of the antenna.

**Figure 8:**
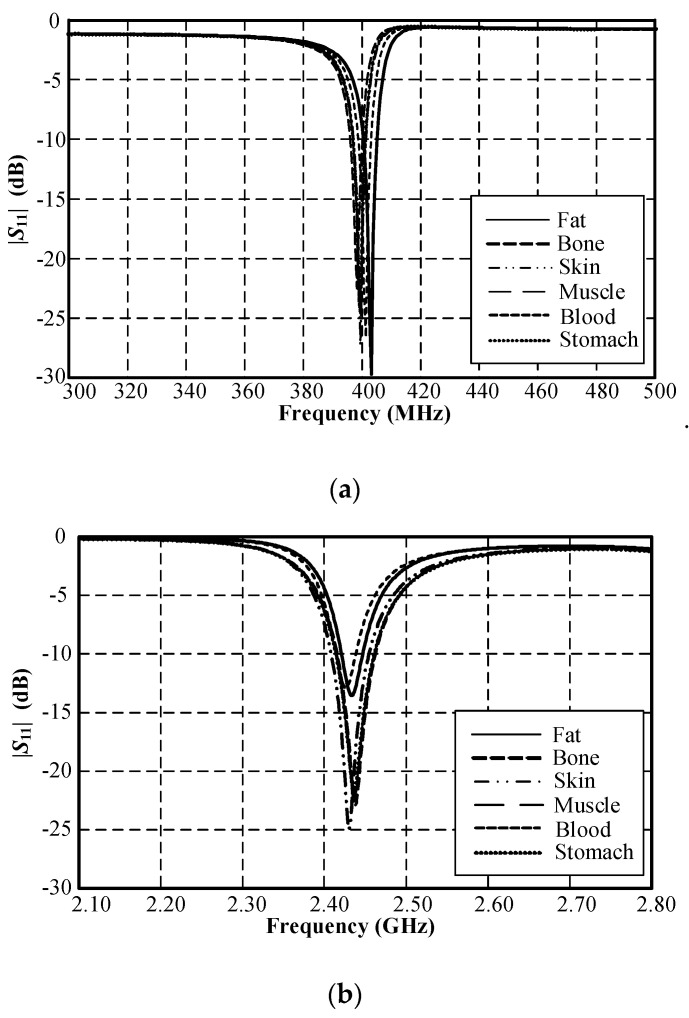
Simulated |*S*_11_| of the MICS/ISM meander-line microstrip antenna encapsulated in a glass pod inside a solid spherical model with a diameter of 50 mm: (**a**) MICS and (**b**) ISM bands.

First of all, the lossy multilayer spherical body-parts model was considered as a single dielectric constant in a spherical model by varying the dielectric constants for each human organ and body part (Table 3). Figure 8 shows the simulated |S_11_| of the MICS/ISM meander-line microstrip antenna encapsulated in a glass pod inside the solid spherical model with a diameter of 50 mm, where the spherical model represented fat, bone, skin, muscle, blood, and the stomach, with varying dielectric constants. The result revealed that the |*S*_11_| varied with the dielectric constants of different layers of organs and body parts. The |*S*_11_| of the MICS band shifted to a higher frequency with an increase in the dielectric constant, while the dielectric constant had a negligible impact on the |*S*_11_| of the ISM bands. High conductivity had a minimal effect on the |*S*_11_| of the MICS band but negatively affected the impedance matching of the ISM band.

**Figure 9:**
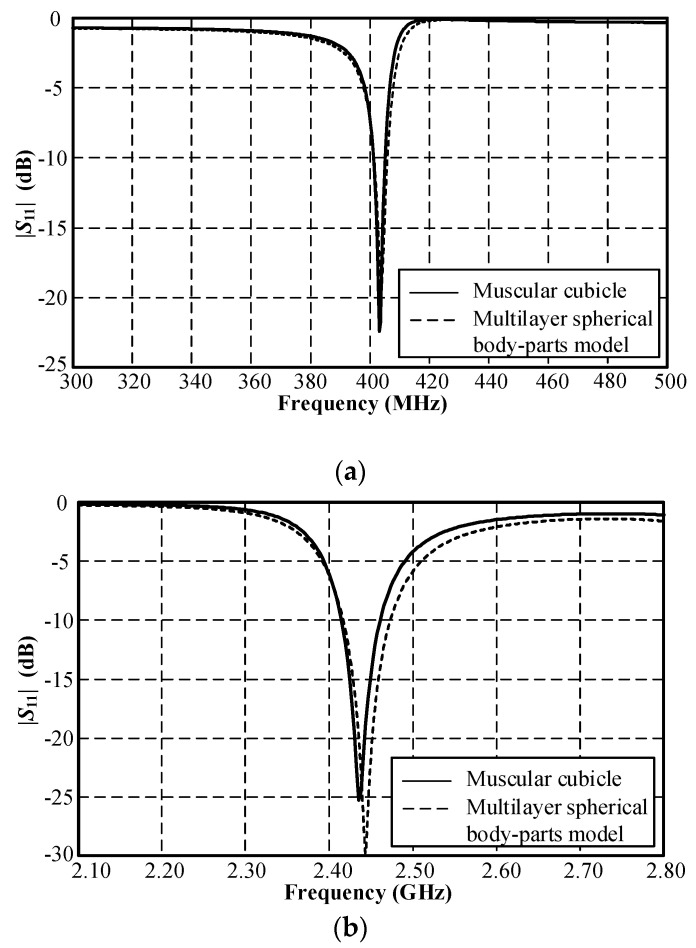
Comparison between the simulated |*S*_11_| of the MICS/ISM meander-line microstrip antenna encapsulated in a glass pod inside the muscular cubicle and in the multilayer spherical body-parts model: (**a**) MICS and (**b**) ISM bands.

Figure 9 compares the simulated |*S*_11_| of the MICS/ISM microstrip antenna encapsulated in a glass pod inside the pseudo-muscular cubicle and in the multilayer spherical body-parts model. The results show the similarity between the |*S*_11_| of the antenna in the muscular cubicle and in multilayer spherical model, indicating the applicability of both models for the antenna simulation.

**Figure 10:**
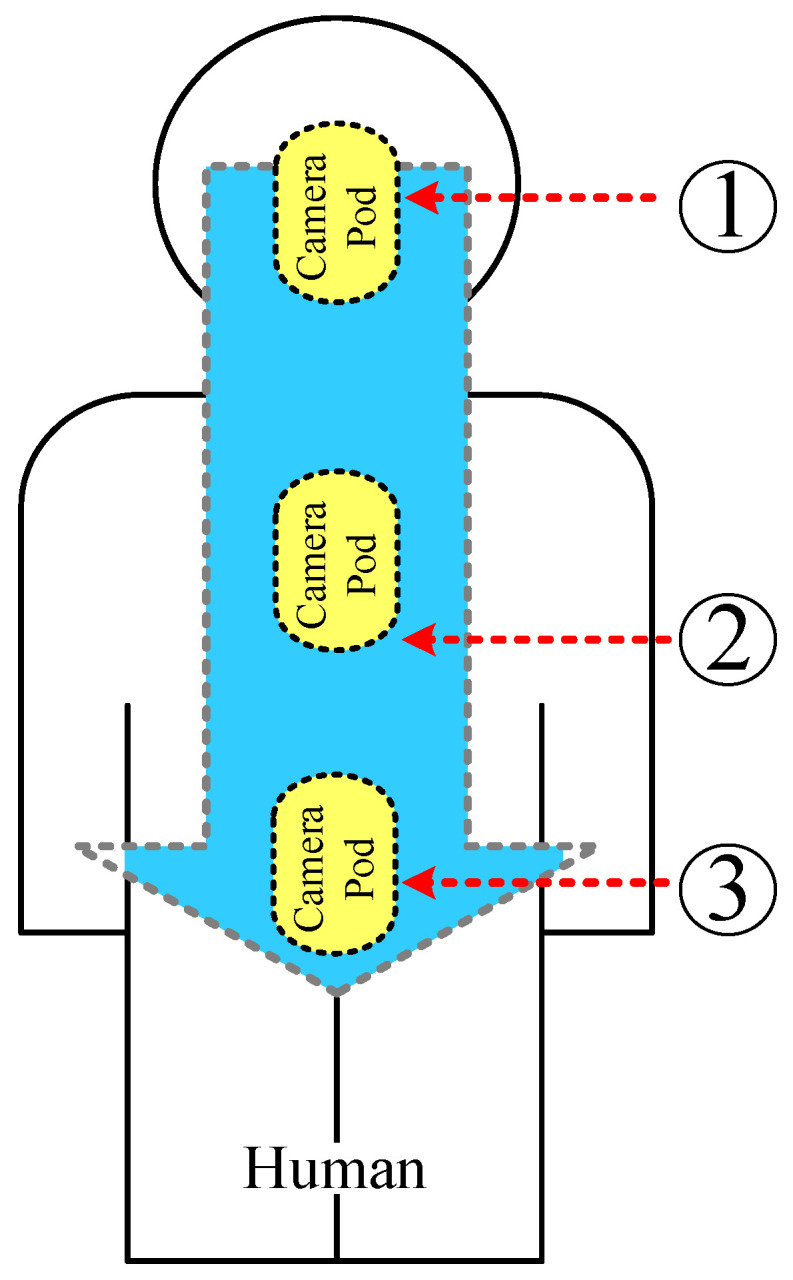
The quasi-human body model with the locations of the pod along the digestive system, where 1, 2, and 3 denote the throat, stomach, and intestine.

**Figure 11:**
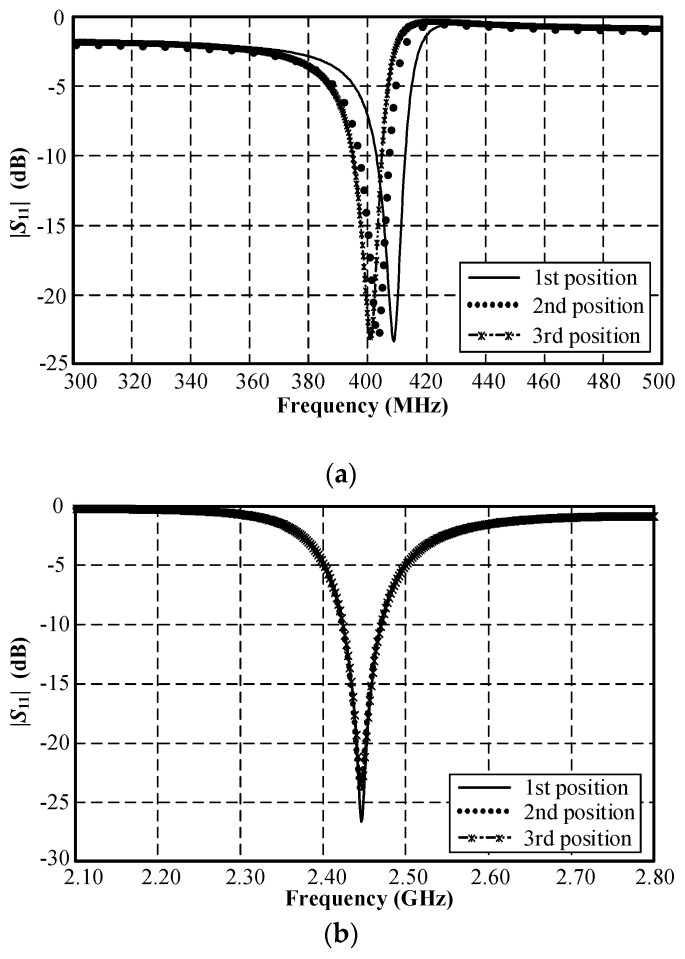
Simulated |S_11_| of the MICS/ISM meander-line microstrip antenna along the digestive tract where the 1st, 2nd, and 3rd positions indicate the throat, stomach, and intestine: (**a**) MICS and (**b**) ISM bands.

In addition, the geometry environment is also affected, which is estimated in Figure 10. Figure 10 depicts the quasi-human body model with the locations of the pod along the digestive tract from the throat through to the intestine. In Figure 11, the simulated |*S*_11_| of the MICS band shifts to a lower frequency as the pod courses along the gastrointestinal tract. However, the impedance-matching (|*S*_11_| ≤ −10 dB) falls inside the MICS band. On the other hand, the location of the pod had a negligible effect on the simulated |*S*_11_| of the ISM band. The physical size of the proposed meander-line microstrip antenna for the quasi-human body was optimized using a global optimization algorithm, namely the genetic algorithm (GA) in CST Microwave Studio [27]. Table 2 shows the optimal physical size of the meander-line microstrip antenna. Due to the high sensitivity of the antenna’s resonant frequency to the flipped-L defected ground plane (Figure 2), the ground-plane parameters (*w*_1_, *l*_2_, and *l*_3_) were optimized with the GA to achieve the resonant frequency at 403 MHz and 2.45 GHz. In the optimization, the GA parameters were as follows: population size, 10; maximum number of iterations, 30; uniform random distribution; mutation rate, 10%; random seed, 5. The GA converged after 10 iterations. The results were compared with the particle swarm optimization (PSO) algorithm in CST Microwave Studio. The comparison of the results from the GA and the PSO is shown in Table 4. These optimizations were employed to find two optimum solutions. The first solution was determined by adjusting the dimension of the antenna (total width and length; *W* × *L*) to achieve the maximum gain. It was obvious that the GA optimization could achieve maximum gains of −22 dBi at 403 MHz and −10.5 dBi at 2.45 GHz, respectively. The maximum gain from the PSO was −21 dBi at 403 MHz and −10 dBi at 2.45 GHz. However, the total sizes of the antenna with the maximum gains from the GA and particle swarm optimizations of 15 × 29 mm and 14 × 31 mm were too large to insert in the glass pod. The second solution was obtained by adjusting the dimensions of the antenna to get the minimum size that was appropriate for the glass pod. The minimum antenna size of 6 × 28 mm was obtained from both the GA and particle swarm optimizations. With this minimum size, the antenna gains from the GA and particle swarm optimizations were −36.04 dBi at 403 MHz and −12.31 dBi at 2.45 GHz, respectively. Moreover, the multi-objective GA and multi-objective PSO were also employed to find the solutions for both the maximum gain and minimum size. The gains were −24 dBi at 403 MHz and −11 dBi at 2.45 GHz for the multi-objective GA optimization and −23 dBi at 403 MHz and −11.5 dBi at 2.45 GHz for the multi-objective PSO. Nevertheless, the dimensions of the antenna from both the multi-objective GA and the multi-objective PSO algorithms of 14 × 29 mm and 15 × 29.5 mm were too large for the glass pod. From these optimization algorithms, it was found that the gain decreased with reductions in the antenna dimensions. Finally, the total size of the antenna was proposed to be 6 × 28 × 1.27 mm (*W* × *L* × *H*). This proposed antenna size is operable in the gastrointestinal tract with commercial OMOM capsules of 13 × 28 mm size [29]. It should be noted that the antenna size can be designed to fit with other smaller commercial capsules at the expense of maximum gain degradation. The proposed antenna can retain impedance matching for human tissue. It can also be robustly integrated with electronic components due to the antenna ground plane protection.

The specific absorption rates at the MICS and ISM bands were determined using CST Microwave Studio [27] with 1 mW input power. In the SAR analysis, the quasi-human body model was discretized into tissues of 1 g and 10 g in volume on average. According to the IEEE C95.1 international standard, the upper limits of the average SAR for the whole body are 0.08 W/kg (for action level) and 0.4 W/kg (for persons in controlled environments) [30]. The simulated maximum 1 g SARs were 0.000429 W/kg at 403 MHz and 0.026202 W/kg at 2.45 GHz. The corresponding 10 g SARs were 0.000209 W/kg at 403 MHz and 0.017021 W/kg at 2.45 GHz. The simulated SARs were substantially below the maximum limits of the international standard, indicating high safety for use inside the human body.

### 3.2. Experimental Results

**Figure 12:**
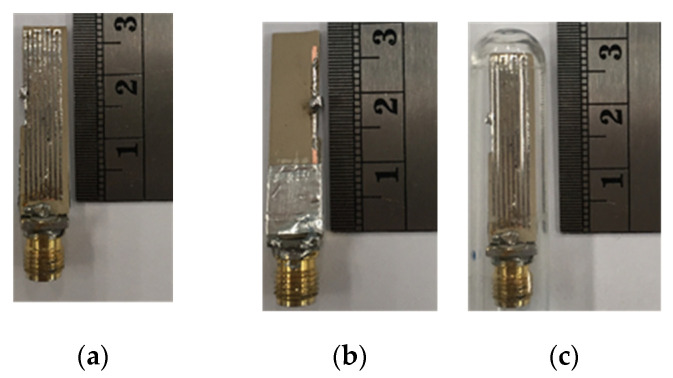
Prototype of the MICS/ISM meander-line microstrip antenna: (**a**) front view, (**b**) rear view, (**c**) in the glass pod.

Figure 12 depicts an antenna prototype fabricated using a Roger RO3210 printed circuit board with a dielectric constant of 10.2. Experiments were carried out with the prototype antenna encapsulated in a glass pod in free space (Figure 12c) and, subsequently, in equivalent liquid mixtures with dielectric constants resembling human tissue [31,32,33]. In the experiments, the equivalent liquid model was used in place of human participants, thereby rendering ethical approval unnecessary.

**Figure 13:**
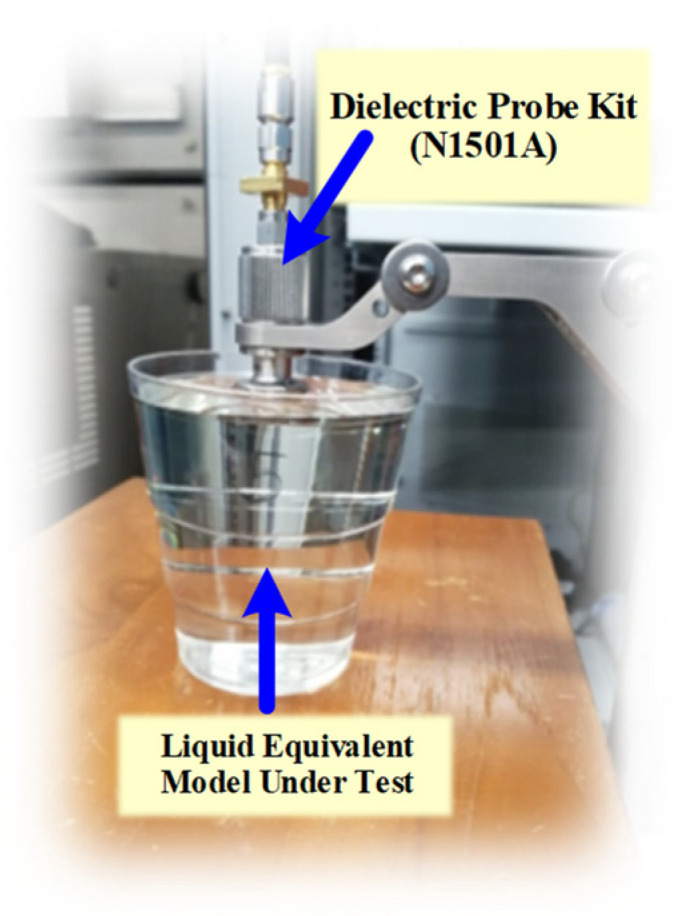
Measurement of the dielectric constants of equivalent liquid using the N1501A dielectric probe kit.

The equivalent liquid model was a mixture of three constituent parts: water, syrup, and salt. Syrup was used to vary the dielectric constant of the water and salt was used to increase the conductivity [31,32]. This method was chosen to obtain permittivity/conductivity relevant to the IEEE head and Federal Communications Commission (FCC) body tissue targets. In addition, the IEEE has provided the target values for head tissue-equivalent liquids in [34,35]. Table 5 lists nine experimental equivalent liquid mixtures obtained by varying syrup, salt, and water concentrations. A dielectric probe kit (N1501A, Keysight Technologies, Santa Rosa, CA, USA) was used to measure the dielectric constants and conductivities of the experimental mixtures, as shown Figure 13.

**Figure 14:**
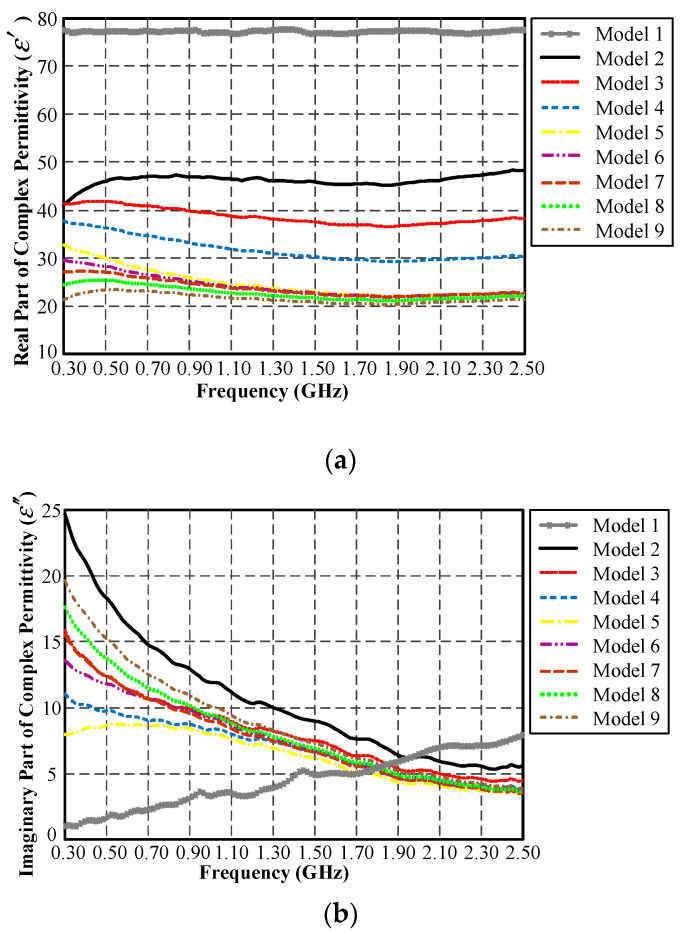
The dielectric constants of nine experimental mixtures of equivalent liquid: (**a**) real part, (**b**) imaginary part.

Figure 14a,b respectively illustrate the real and imaginary parts of the dielectric constants of nine experimental equivalent liquid mixtures. In Figure 14a, mixture 1 (water) exhibits the highest dielectric constant, and the dielectric constant decreases with increases in the syrup concentration. Meanwhile, the conductivity can be seen to increase with increases in the salt concentration (Figure 14b) [32].

**Figure 15:**
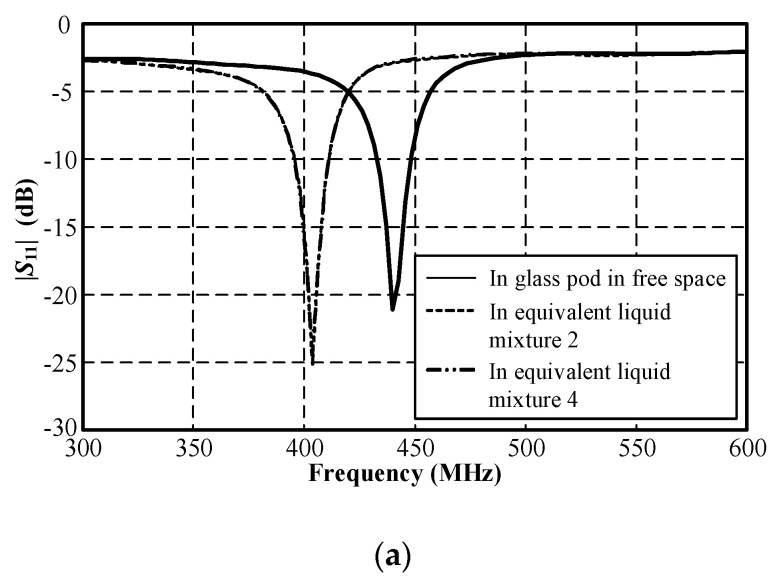

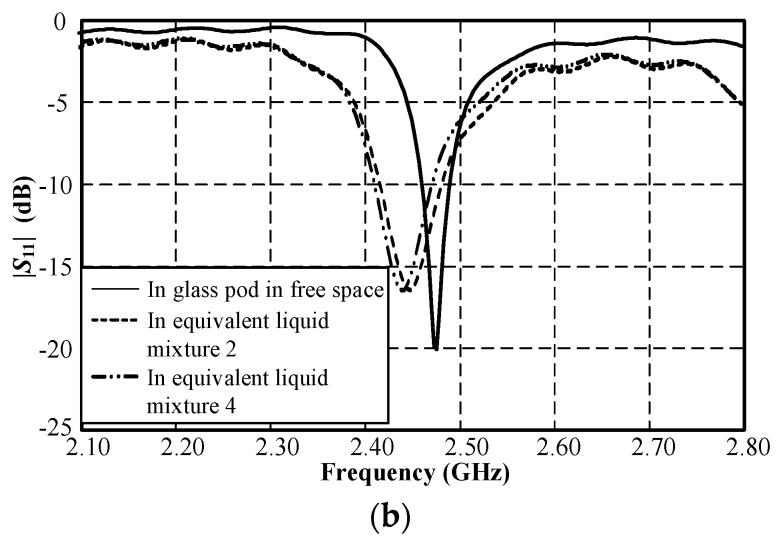
Measured |*S*_11_| of the MICS/ISM meander-line microstrip antenna encapsulated in a glass pod in free space and in equivalent liquid mixtures 2 and 4: (**a**) MICS and (**b**) ISM bands.

**Figure 16:**
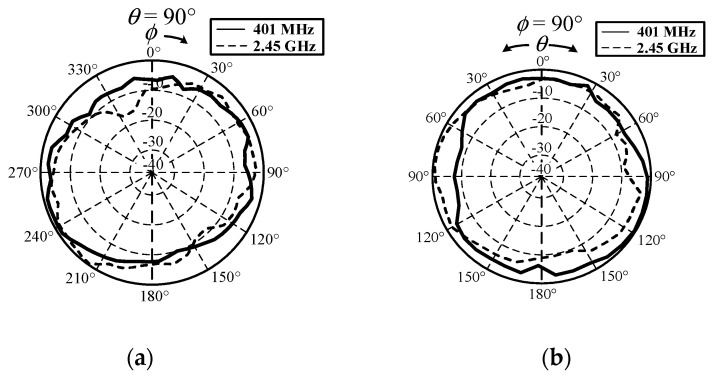
Measured radiation patterns of MICS/ISM meander-line microstrip antenna in equivalent liquid mixtures 2 and 4: (**a**) *xy* and (**b**) *yz* planes.

Figure 15 compares the measured |*S*_11_| of the MICS/ISM meander-line microstrip antenna encapsulated in a glass pod in free space and in equivalent liquid mixtures 2 and 4 (Table 5). In these experiments we selected the equivalent liquid mixtures 2 and 4 because their dielectric constants closely resembled those of human body parts with dielectric constants between 30 and 48, as shown in Figure 14. Figure 16 depicts the measured *xy*- and *yz*-plane radiation patterns of the MICS/ISM meander-line antenna in equivalent liquid mixtures 2 and 4. The radiation has a quasi-omnidirectional pattern. The measured impedance bandwidths (|*S*_11_| ≤ −10 dB) for the MICS and ISM bands were 398–407 MHz and 2.41–2.48 GHz, with corresponding antenna gains of −38 dBi and −13 dBi.

**Figure 17:**
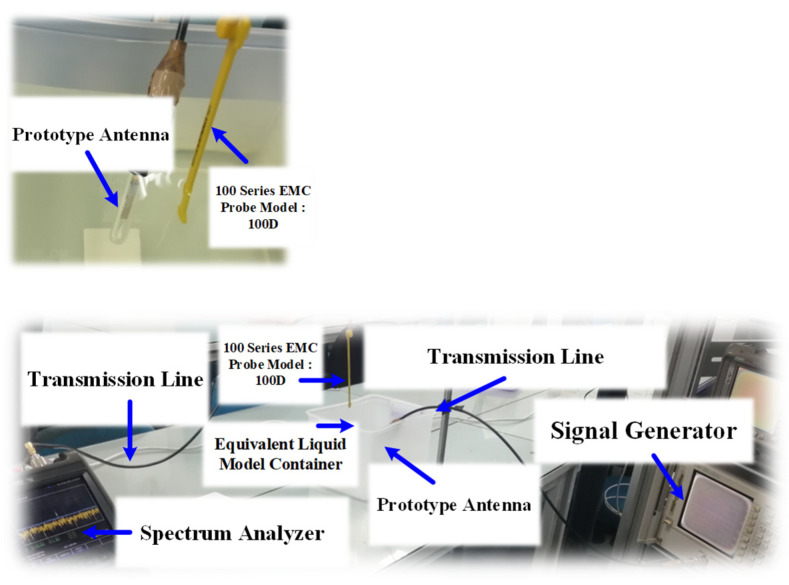
SAR measurement setup in equivalent liquid mixtures with the antenna prototype encapsulated in a glass pod.

Figure 17 depicts the SAR measurement setup in equivalent liquid mixtures using a 100 series EMC probe 100D model [36]. The probe reading (*P*_out_) was converted into electric field strength using Equation (1) [36], where *P*_out_ is the output power from the probe (dBm), *F* is the operating frequency (MHz) (i.e., 403 MHz and 2450 MHz), and *E* is the electric field strength (V/m).

(1) $$P_{out}=-113.2+20\log(E)+20\log(F)$$

The distance between the MICS/ISM antenna prototype and the EMC probe had to be greater than $a^{2}/\lambda$, where a is the largest dimension of the prototype antenna. The SAR measurement was carried out in a continuous fashion for a total time of 6 min using the max-hold function in the spectrum analyzer (Fieldfox Handheld Spectrum Analyzer) and the maximum electric field strength (*E_max_*) was determined. The specified averaging time of 6 min was an average of the time required for the maximum permissible values of the RF field strength or power density, following [34]. The SAR was then calculated using Equation (2) [34,35]:

(2) $$SAR=\frac{1}{\rho }\omega \varepsilon_{0}\varepsilon^{\prime \prime }E_{\max}^{2}$$

where ρ is the mass density in kg/m^3^, ω is the radian frequency, $\varepsilon_{0}$ is the permittivity of free space (8.854 × 10^−12^ F/m), $\varepsilon^{\prime \prime }$ is the imaginary part of the complex relative permittivity, σ is the conductivity in S/m, and *E_max_* is the maximum electric field strength.

The measured SAR of equivalent liquid mixture 2 was 0.0054 W/kg at 403 MHz and 0.000587 W/kg at 2.45 GHz. For equivalent liquid mixture 4, the corresponding SARs were 0.0026 W/kg and 0.000421 W/kg. The measured SARs were substantially below the maximum safety limits of the IEEE C95.1 international standard, in which the upper limits of average SARs for the whole body are 0.08 W/kg (for action level) and 0.4 W/kg (for persons in controlled environments) [30].

## 4. Conclusions

This study proposed a MICS/ISM meander-line microstrip antenna encapsulated in an oblong-shaped pod for use in diagnoses of the gastrointestinal tract. The proposed microstrip antenna is operable in MICS and ISM bands and its structure consists of a meander-line radiating patch, a flipped-L defected ground plane, and a loading resistor to reduce the antenna’s total size. Simulations in a muscular cubicle and multilayer spherical model were carried out to determine the optimal physical size of the antenna. In a quasi-human body simulation, the impedance bandwidths (|*S*_11_| ≤ −10 dB) for the MICS and ISM bands were 400–406 MHz and 2.41–2.47 GHz, with respective antenna gains of −36.04 dBi and −12.31 dBi. The simulated radiation had a quasi-omnidirectional pattern. For verification, an antenna prototype was fabricated and experiments conducted in equivalent liquid mixtures with dielectric constants resembling those of human tissue. The measured impedance bandwidths (|*S*_11_| ≤ −10 dB) for the MICS and ISM bands were 398–407 MHz and 2.41–2.48 GHz. The corresponding antenna gains were −38 dBi and −13 dBi, with quasi-omnidirectional radiation patterns. The simulation and the measured results were thus in good agreement. The measured SARs were between 0.0026–0.0054 W/kg at 403 MHz and 0.000421–0.000587 W/kg at 2.45 GHz, which are substantially below the maximum safety limits of the IEEE C95.1 standard. The MICS/ISM microstrip antenna encapsulated in an oblong-shaped pod can potentially be used as an ingestible diagnostic tool for real-time gastrointestinal tract diagnosis.

## Figures and Tables

**Table 1:** Comparison of existing multiband antenna schemes for medical-related applications and the scheme proposed in the present article.

Antenna	Dimension (mm)	Gain	Frequency Band
[15]	25 × 34 × 2.5	−33 dBi at 1.42 GHz and −24 dBi at 2.4 GHz	WMTS and ISM
[16]	15 × 15 × 1.92	−12.25 dBi at 403 MHz and −12.4 dBi at 2.45 GHz	MICS and ISM
[17]	19 × 30 × 1.6	−32 dBi at 433 MHz, −11.5 dBi at 1433 MHz and −13 dBi at 2.4 GHz	MICS, WMTS, and ISM
Proposed Antenna	6 × 28 × 1.27	−38 dBi at 403 MHz and −13 dBi at 2.45 GHz	MICS and ISM

**Table 2:** Parameters of the proposed MICS/ISM meander-line microstrip antenna.

Parameter	Description	Initial Physical Size (mm)	GA-Optimized Physical Size (mm)
*W*	Total width	6	6
*L*	Total length	28	28
*w*_1_	Width of defected ground plane	5.5	5.0
*l*_1_	Position of loading resistor (2 Ω)	11	11
*l*_2_	Length of defected ground plane	26	25
*l*_3_	Length of flipped L	9	8.5
*t*	Thickness of substrate	1.27	1.27
*t*_1_	Thickness of meander line	0.25	0.25
*s*	Distance between meander lines	0.5	0.5

**Table 3:** The conductivities and dielectric constants of human organs and body parts [28].

Body Parts	Conductivity (σ): S/m		Dielectric Constant (*ε*_r_)	
	*f* = 403 MHz	*f* = 2.45 GHz	*f* = 403 MHz	*f* = 2.45 GHz
Stomach	1.00	2.21	67.48	62.15
Small intestine	1.90	3.17	66.14	54.42
Blood	1.34	2.54	64.18	58.26
Bone	0.23	0.80	22.44	18.56
Muscle	0.79	1.74	57.12	52.72
Fat	0.04	0.10	5.57	5.27
Skin	0.68	1.46	46.78	38.00

**Table 4:** Comparison of the optimization algorithms.

Optimization Algorithm	GA		Multi-Objective GA	PSO		Multi-Objective PSO
Optimization Goal	Maximum gain	Minimum size	Maximum gain and minimum size	Maximum gain	Minimum size	Maximum gain and minimum size
Dimensions (mm)	15 × 29	6 × 28	14 × 29	14 × 31	6 × 28	15 × 29.5
Antenna gain	−22 dBi at 403 MHz, −10.5 dBi at 2.45 GHz	−36.04 dBi at 403 MHz, −12.31 dBi at 2.45 GHz	−24 dBi at 403 MHz, −11 dBi at 2.45 GHz	−21 dBi at 403 MHz, −10 dBi at 2.45 GHz	−36.04 dBi at 403 MHz, −12.31 dBi at 2.45 GHz	−23 dBi at 403 MHz, −11.5 dBi at 2.45 GHz

**Table 5:** The concentrations of syrup, salt, and water in nine experimental mixtures of equivalent liquid.

Mixture	Syrup	Salt	Water
1	0%	0%	100%
2	20%	1%	79%
3	30%	1%	69%
4	40%	1%	59%
5	50%	1%	49%
6	50%	2%	48%
7	50%	3%	47%
8	50%	4%	46%
9	50%	5%	45%

## Data Availability

The data used to support the findings of this study are available from the corresponding author upon request.
